# Tracking transcriptomic responses to endogenous and exogenous variation in cetaceans in the Southern California Bight

**DOI:** 10.1093/conphys/coz018

**Published:** 2019-05-15

**Authors:** Marisa L Trego, Andrew Whitehead, Nicholas M Kellar, Morgane Lauf, Rebecca L Lewison

**Affiliations:** 1 Department of Biology, San Diego State University, San Diego, CA, USA; 2 Department of Environmental Toxicology, University of California Davis, Davis, CA, USA; 3 Ocean Associates, Inc., under contract to the Southwest Fisheries Science Center, National Marine Fisheries Service, National Oceanic and Atmospheric Administration, Arlington, VA, USA

**Keywords:** Ecotype, marine mammal, temperature, transcriptomics

## Abstract

Marine wildlife populations are adapted to survive in highly dynamic environments. However, identifying the effects of endogenous versus exogenous variables on marine mammal physiology remains a substantial challenge in part because of the logistical constraints that limit the collection of physiological data in free-ranging animals. Measuring genome-wide gene expression is one minimally invasive method that can be used to elucidate how free-ranging cetaceans’ physiological responses shift with changing environmental conditions or demographic states, i.e. reproductive status and maturity. We identified transcriptomic differences among bottlenose dolphins (*Tursiops truncatus*) from the Southern California Bight using RNAseq data from the skin of 75 individuals to examine gene expression associated with sex, pregnancy status, sea surface temperature, geographic location and ecotype. We identified transcriptomic variation between two genetically distinct ecotypes as well as variation related to environmental conditions among groups that exhibit little evidence of genetic divergence. Specifically, we found differential expression of genes associated with structural development, cellular starvation and immune response. Sex and pregnancy status explained a small proportion of the observed variation, in contrast to sea surface temperature, which explained a substantial amount of transcriptomic variation. However, these measured variables did not account for all of the differential expression observed between ecotypes and among geographically distinct groups. Additional research is needed to identify other endogenous or exogenous factors that may be contributing to observed transcriptomic differences among ecotypes.

## Teaser

We investigated genome-wide gene expression associated with sex, pregnancy status, sea surface temperature, geographic location, and ecotype in bottlenose dolphins from the Southern California Bight. Our findings suggest that gene expression in cetacean skin is affected by changes in oceanographic temperature and differs between genetically distinct ecotypes.

## Introduction

Marine mammals inhabit highly dynamic environments where they are regularly exposed to a variety of conditions and stressors, both natural and human mediated (Becker *et al.*, 2010; Carmichael *et al.*, 2012; Forney *et al.*, 2017), which have been linked to changes in reproductive health (Hansen, 2009; Liptrap, 1993; Rensis and Scaramuzzi, 2003; Tilbrook *et al.*, 2000) and survival. To identify the mechanisms that connect environmental shifts to individual physiological changes, which can ultimately affect population viability (Liptrap, 1993), requires the ability to monitor changes in physiology in wild individuals. However, detecting physiological changes in wild marine mammals *in situ* remains a substantial challenge due to the logistical constraints that limit the ability to collect physiological data from free-ranging animals.

Skin tissue collected through biopsies is one sample type that has been used to monitor physiology *in situ* in many marine mammal populations (Fossi *et al.*, 1992; Mollenhauer *et al.*, 2009; Van Dolah *et al.*, 2015). Marine mammal skin serves as the primary protective barrier from the external environment and maintains internal homeostasis (Elias, 1988; Elias *et al.*, 1987) and is one of the only tissue types widely accessible for genetic and physiological analysis (Kellar *et al.*, 2013, 2006). Skin analyses have been used to provide a mechanistic understanding of interactions between cetaceans and their environment (Neely *et al.*, 2017; Van Dolah *et al.*, 2015). Much of this work has centred upon on identifying *in vitro* cellular responses to contaminant exposure (Fossi *et al.*, 2010, 1992; Panti *et al.*, 2011) and has yet to be applied widely to physiological questions in wild populations.

Gene expression analysis is a valuable tool that is commonly utilized to examine how wild organisms respond to changing environmental conditions and stressors (Evans and Hofmann, 2012; Howarth and Ougham, 1993; Piña *et al.*, 2007). Gene expression analyses aim to quantify changes in RNA transcripts that relate to different conditions. By identifying the functions of the genes being differentially regulated, it is possible to infer how downstream physiological processes are changing in relation to specific factors (Ashburner *et al.*, 2000). Gene expression is shaped both by external factors (Alvarez *et al.*, 2015), such as temperature (Huang *et al.*, 2011; Srikanth *et al.*, 2017) and contaminant exposure (Veldhoen *et al.*, 2012; Whitehead *et al.*, 2012), but can also differ based on intrinsic parameters, such as sex (Mancia *et al.*, 2015; Morey *et al.*, 2016) or adaptive variation (Cammen *et al.*, 2015a, 2015b; Whitehead and Crawford, 2006).

One of the preferred methods for gene expression analyses is RNAseq as it examines genome-wide gene expression (i.e. whole transcriptome expression) and does not require *a priori* characterization of target genes providing a less biased assessment of gene expression compared to previous technologies (e.g. microarrays). RNAseq has been demonstrated to be a robust approach for monitoring marine mammals’ physiological responses to temperature changes (Morey *et al.*, 2016; Neely *et al.*, 2017; Van Dolah *et al.*, 2015), contaminant exposure (Buckman *et al.*, 2011; Mollenhauer *et al.*, 2009; Neely *et al.*, 2017) and stress (Khudyakov *et al.*, 2017, 2015). However, some of these studies have been limited by the number of genes included (Buckman *et al.*, 2011; Mollenhauer *et al.*, 2009), by nature of being conducted *ex vivo* rather than *in situ* (Fossi *et al.*, 2010; Godard-Codding *et al.*, 2011; Lunardi *et al.*, 2016), and by using blood, a sample type that is typically unavailable from wild, free-ranging marine mammals (Morey *et al.*, 2016). This is one of the first studies to apply skin transcriptomics in health assessments of *Tursiops truncatus* populations on the West Coast of the United States (see Trego *et al*., 2019).

Marine mammals in the Southern California Bight (SCB) inhabit a particularly dynamic environment (Bograd and Lynn, 2003; Winant and Bratkovich, 1981). The SCB has recently experienced an increase in oceanographic anomalies with uncharacteristically warm temperatures across the north Pacific starting in 2014 (Peterson *et al.*, 2017; Zaba and Rudnick, 2016). Referred to as ‘The Blob’, these abnormally warm water masses in the eastern Pacific have been associated with a reduction in primary productivity (Gómez-Ocampo *et al.*, 2017) and larval settlement (Basilio *et al.*, 2017) that likely impacted food availability for higher trophic level organisms (Peterson *et al.*, 2017). This warming has been linked to a decline in foraging quality, reproductive success and body condition in pinnipeds off the coast of Baja California, Mexico (Elorriaga-Verplancken *et al.*, 2016).

SCB marine mammals also experience a wide range of environmental conditions that are influenced by anthropogenic activities. The SCB region is characterized by a high concentration of anthropogenic contaminants that are known to be toxic (Chapman, 1996; Dodder *et al.*, 2012; Young, *et al.*, 1977; Young *et al.*, 1976) as well as a variety of other stressors thought to impact physiological health, including noise from naval activity and high shipping traffic (Forney *et al.*, 2017; Redfern *et al.*, 2017). An increase in the number and magnitude of stressors that marine mammals are exposed to could pose a health risk, particularly to those individuals or stocks in close proximity to urban areas.

The bottlenose dolphin (*T. truncatus*) is one of the resident marine mammals in the SCB with several confirmed and putative ecotypes, i.e. genetically distinct varieties or populations within a species that are adapted to specific environments (Valentine, 1949). The coastal ecotype is composed of ~450–500 individuals that inhabit a narrow zone (typically 1-km wide) along the Pacific coast from San Francisco to Baja California, Mexico (Hwang *et al.*, 2014; Caretta *et al.*, 2017). The offshore ecotype has an estimated 1000 to 2000 individuals and is distributed across a much larger range centred primarily on the Channel Islands, typically farther than 1 km from the mainland coastline (Caretta *et al.*, 2017). Data suggests that the two ecotypes do not overlap, despite absence of physical barriers. Morphological differences between the ecotypes suggest evidence of adaptation to different diets and environmental features, as the coastal ecotype is primarily located on the more shallow, narrow continental shelf and the offshore ecotype is more often found in areas with deeper bathymetry (Perrin *et al.*, 2011). A third putative offshore ecotype primarily located in the Eastern Tropical Pacific (referred to as ETP) is believed to be a distinct population from the coastal and offshore ecotypes, though extensive genetic analyses have not been conducted. Due to its small size and close proximity to highly urbanized coastal areas, there is particular interest in the health of individuals in the coastal *T. truncatus* ecotype as compared to other ecotypes (Trego *et al*., 2019).

**Figure 1:**
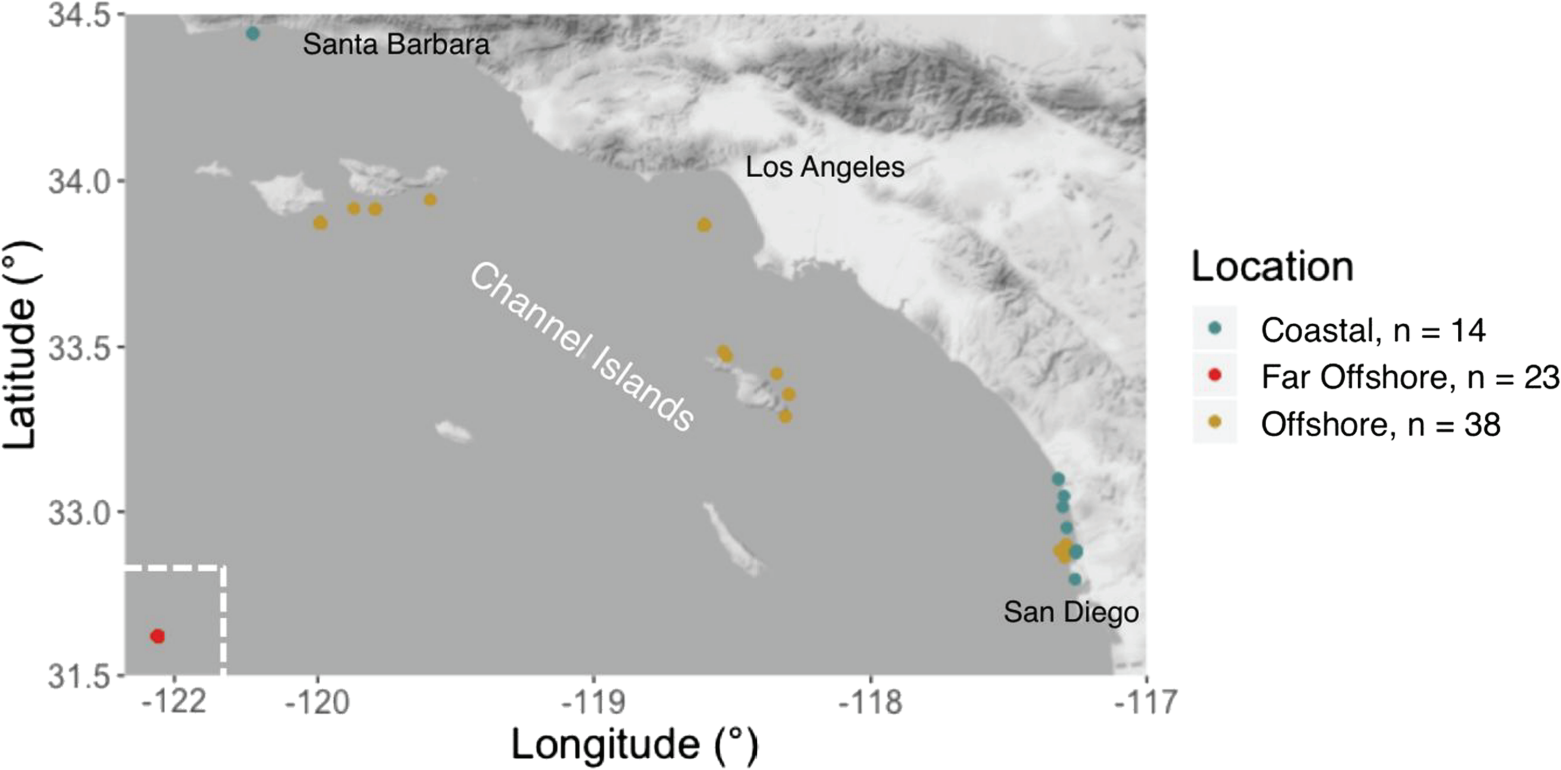
A map of the sampling area and the designated sampling locations for individuals included in this study.

This study considers how cellular activity in cetacean skin responds to environmental variability, specifically sea surface temperature, within the context of other factors including sex, pregnancy status, geographic location and ecotype. We used RNAseq to profile transcriptomes from skin tissue samples of 75 *T. truncatus* individuals from three ecotypes within the SCB collected between 2012 and 2016. This research provides novel information on how physiological processes may differ among dolphin ecotypes, as well as demographic and environmental conditions in the SCB. Our research highlights the importance and utility of monitoring transcriptomic biomarkers in marine mammal physiology.

## Materials and methods

### Sample description and collection

Biopsies composed of skin and blubber were collected from 75 *T. truncatus* individuals, 35 females and 40 males, between 2012 and 2016 via crossbow. All biopsies were collected under NOAA permit #14097-06 and approved by the Southwest Pacific Islands IACUC (SWPI2013-06 and SWPI2015-03A). Of the samples collected, 14 were from a coastal ecotype that resides within the SCB, 38 were collected from the offshore ecotype and 23 from a far offshore group of unknown origin (Fig. 1). Ecotype was designated based on established analyses of mitochondrial markers for coastal and offshore ecotype combined with sampling location. Single nucleotide polymorphism (SNP) analyses were conducted to classify the far offshore group (see Data analysis: Ecotype designation). Sex was confirmed by genetic analysis as in Kellar *et al.* (2014). Samples were flash frozen in liquid nitrogen upon collection. Though blubber can be used for transcriptomic analysis, we focused our gene expression profiling in skin because blubber was prioritized for other uses by us and our collaborators. A subsample of skin for RNAseq analysis was subsequently stored in RNAlater and kept at −20°C until RNAseq analysis. The remaining blubber tissue was stored at −80°C for hormone analysis.

### Hormone analysis

To assess pregnancy status, we quantified progesterone in the blubber of all female samples. Between 80 to 150 mg of blubber was homogenized and progesterone extracted according to methods published in Kellar *et al.* (2006) and Trego *et al*. (2013) Tissue was homogenized using an Omni BeadRuptor (Omni International, Kennesaw, GA, USA) in metal tubes (BioSpec Products, Bartlesville, OK, USA) with garnet and ceramic beads. Progesterone was then isolated from the homogenate using a biphasic solvent extraction and analysed with enzyme immunoassay kits for progesterone (ADI-900-011, Enzo Life Sciences, Farmingdale, NY, USA). Each extraction included a set of non-spiked and spiked controls to estimate extraction efficiency. Efficiency was estimated by calculating the percent of progesterone recovered in controls spiked with known amounts of hormone. Blubber hormone concentrations were lipid corrected. Pregnancy was diagnosed according to blubber progesterone cutoffs derived from data in Kellar *et al*. (2017), in which live-caught *T. truncatus* individuals had pregnancies confirmed via ultrasound. Any individual with a blubber progesterone level <14 ng/g was assumed to not be pregnant, those >40 ng/g were presumed pregnant and any in between were considered ambiguous and removed from the analysis (see Table S1).

### RNAseq data collection

We used skin to examine transcriptomic variation among individuals within our sample set. Skin stored in RNAlater was extracted for RNAseq with Qiagen RNeasy Mini kits (#74104, Qiagen Inc., Valencia, CA, USA). A 15- to 20-mg cross-section of skin, predominantly composed of epidermis with some dermal papillary tissue, was homogenized in Qiazol with **β**-mercaptoethanol using an Omni BeadRuptor. Homogenate was then processed according to the protocols provided by the Qiagen RNeasy kit. RNA quality was analysed using an Agilent Bioanalyzer (Agilent, Santa Clara, CA, USA) and quantification with a Qubit fluorometer (Invitrogen, Thermo Fisher Scientific, Waltham, MA, USA). All samples had RNA integrity numbers (RINs) over 7 with the exception of six individuals with values between 6.2 and 6.9, which were still included in this analysis due to the small sample set and reasonable bioanalyser profiles. There was no apparent difference in expression in individuals with lower RINs.

Libraries were prepared for RNAseq analysis using NEBNext Ultra Directional RNA Library Prep Kit for Illumina (#E7420S) and indexed with NEBNext Multiplex Oligos for Illumina (#E7600S, New England Biolabs, Ipswich, MA, USA). Library quality was checked on an Agilent Bioanalyzer and quantified via Qubit fluorometer. Final libraries were cleaned, pooled and run on an Illumina HiSeq 3000/4000 platform at the UC Davis Genome Center (100-bp paired-end reads) at a depth of between 15 and 20 million paired-end reads per sample.

**Figure 2:**
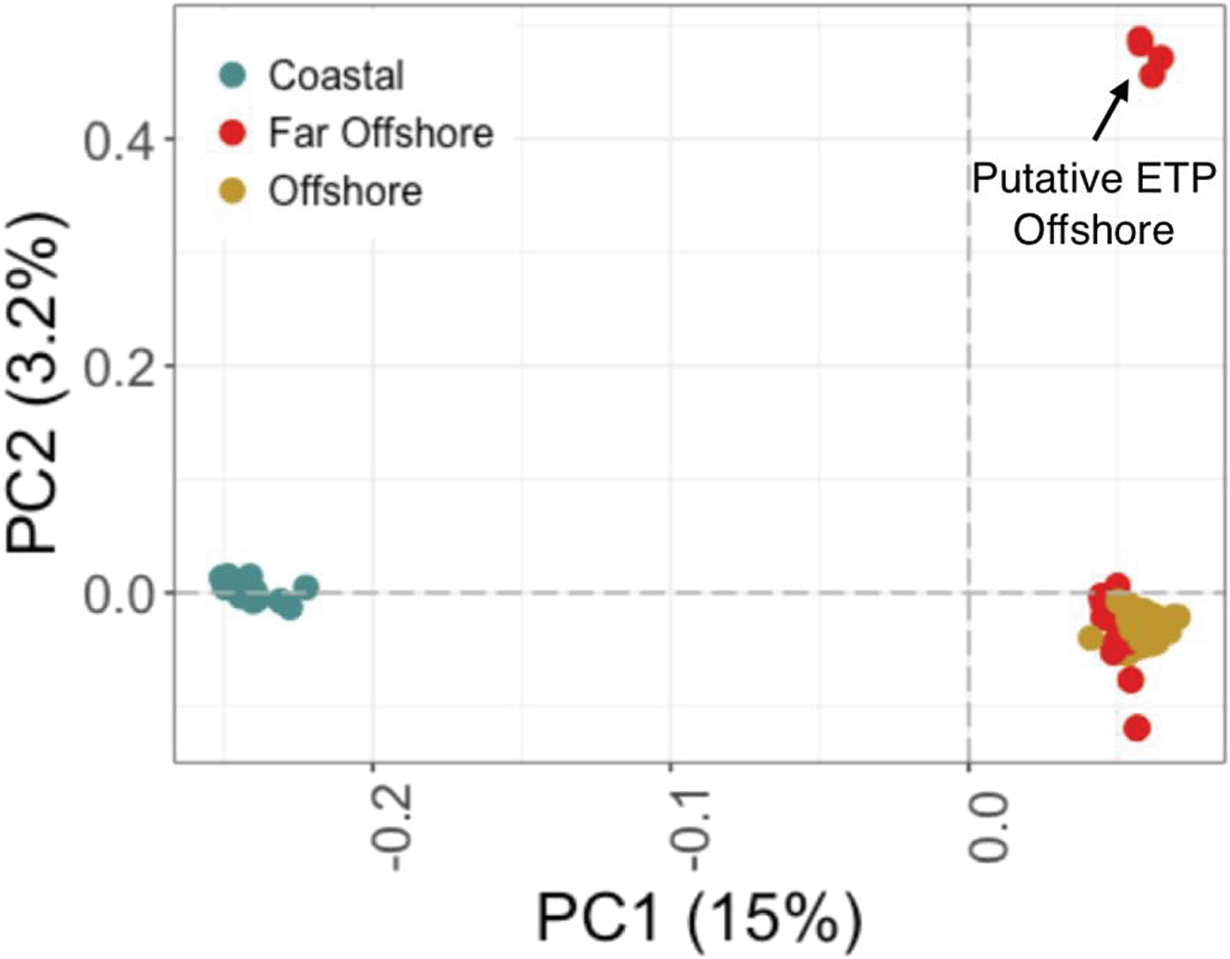
A PCA of the SNPs demonstrating genetic clusters of the three geographic locations.

## Data analysis

All analyses were conducted on a RedHat Linux system, an XSEDE Jetstream instance or in R version 3.4.4 (R Core Team, 2017). Mean sea surface temperature for each sampling date and location was obtained from the erdGAssta (Maturi *et al.*, 2004; Merchant *et al.*, 2005; Wu *et al.*, 1999) dataset available from ERDDAP (Simons, 2017) with the Xtractomatic (3.4.2; Mendelssohn, 2018) R package. All sampling dates were a 1-day composite with the exception of two sampling dates that only had 3-day composites available. The composite was estimated across a 0.1-degree square area around the sampling latitude and longitude.

### Ecotype designation

Ecotype designation for the coastal and offshore was based on established mitochondrial analyses (Lowther-Thieleking *et al.*, 2015). To confirm ecotype, we also identified SNPs in our RNAseq data in order to confirm population identification and determine genetic distance between the far offshore individuals and the recognized coastal and offshore ecotypes. Polymerase chain reaction (PCR) duplicates were removed from samples after mapping to the genome (turTru1) with TopHat2 (v2.2.1; Kim *et al.*, 2013), and the cleaned data were then run in Freebayes (Garrison and Marth, 2012) to identify variants. We filtered the data with VCFtools (Danecek *et al.*, 2011) to identify SNPs that were present in at least 95% of individuals, had a quality score of 30 or above and had between 10× and 100× coverage per individual. A principal components analysis (PCA) was used to visualize clustering of samples according to presumed ecotype with the SNPrelate package in R (Zheng *et al.*, 2012). Weir and Cockerhams Fst (Weir and Cockerham, 1984) was calculated between putative ecotypes as an estimate of genetic distance with VCFtools. Based on these data, the far offshore group appeared to be composed of two potential stocks: the majority was genetically indistinguishable from the offshore ecotype with the exception of four individuals that exhibited genetic separation from the rest of the group (Fig. 2). These four individuals could potentially be from a *T. truncatus* stock from the ETP, and we refer to them genetically as putative ETP individuals. We used the genetic clusters identified here to categorize individuals by ecotype for differential expression analysis.

**Figure 3:**
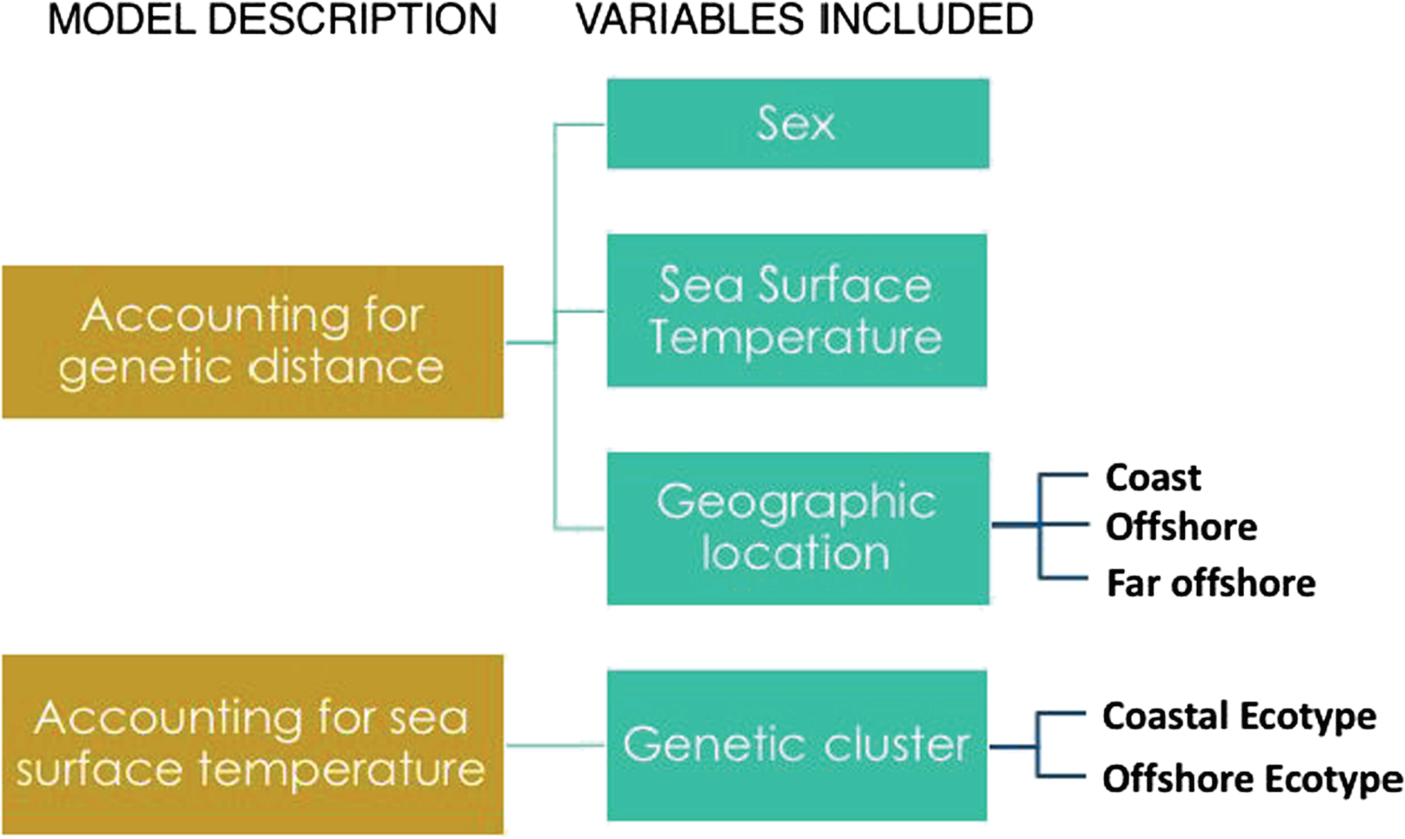
A schematic of the DESeq2 models that were used to investigate differential expression.

### Transcriptome assembly and differential expression

In order to evaluate differential expression, we assembled and annotated a transcriptome, then counted all reads aligning to the annotated genes. RNAseq data were trimmed and quality filtered with Trimmomatic (v0.36; Bolger *et al.*, 2014). We then performed the genome-guided assembly with the Ensembl *T. truncatus* genome (turTru1) using TopHat2 (v2.2.1; Kim *et al.*, 2013) and cufflinks (v2.2.1; Trapnell *et al.*, 2012). Transcriptome completeness was assessed with benchmarking universal single-copy orthologs (BUSCO, v3; Simão *et al.*, 2015; Waterhouse *et al.*, 2018). The assembled transcriptome was then annotated using dammit (Scott, 2016) on an XSEDE Jetstream instance (Towns *et al.*, 2014; Stewart *et al.*, 2015). For differential expression analyses, we first converted protein IDs from dammit annotation to gene name when possible. All trimmed reads were then mapped to the new transcriptome with Salmon (v0.9.1; Patro *et al.*, 2017). Aligned transcripts were counted by gene name with tximport (1.6.0; (Soneson *et al.*, 2015). Then differential expression was evaluated with DEseq2 (v1.18.1; Love *et al.*, 2014).

We built two models to examine differential expression: one to examine differential expression not explained by genetic distance and another to identify differential expression between genetically distinct ecotypes (depicted in Fig. 3). First, to disentangle genetic distance from differential expression due to other factors, we built a model including the first principal component from the SNP PCA as a proxy for genetic distance. Models in DEseq2 are designed such that they consider variation in a preceding variable prior to assessing variance of the subsequent variable. After accounting for variation due to genetic distance (i.e. a proxy for genetic distance was the first variable in the DEseq2 model), we identified differential expression related to sea surface temperature, sampling location (coastal, offshore or far offshore) and sex. A second model was built to examine differences between genetically distinct ecotypes. After accounting for variance due to mean sea surface temperature, we identified differential expression between coastal and offshore ecotypes where the offshore group included all individuals from the far offshore group with genetic similarities with the offshore individuals. Final results for each factor were assessed after log fold change (LFC) shrinkage. One additional model was used to examine differential expression related to pregnancy within the offshore ecotype, which was the only group with a sufficient number of individuals predicted to be pregnant.

The data were filtered to remove all genes with zero counts for more than 45 individuals, and significantly differentially expressed genes were identified as those with false discovery rate adjusted *P*-values <0.05 and a log 2-fold change above or below one (i.e. an absolute fold change >2). The LFC threshold was not used for mean sea surface temperature because fold change with linear predictors in DESeq2 measures the rate of change with sea surface temperature (i.e. akin to a slope) rather than the absolute fold change. For differential expression relative to pregnancy, only 18 individuals were tested so criteria were altered to remove all samples with more than 11 individuals with zero counts. A PCA was used for data visualization and gene clustering.

### GO enrichment and weighted gene co-expression

To relate differential gene expression to biological functions, gene ontology (GO) terms (universal terms that connect genes to biological, molecular and cellular activities) were acquired using *T. truncatus*, *Bos taurus* and *Homo sapiens* Ensembl Biomart databases and the biomaRt R package (2.34.2; Durinck *et al.*, 2009, 2005). GO enrichment analyses were performed on all genes with an adjusted *P*-value less than or equal to 0.05 with TopGO (Alexa and Rahnenführer, 2009). We used a classic Fishers test to determine GO term enrichment for each pairwise ecotype comparison and for mean sea surface temperature. We included tests for each GO component: biological processes, cellular components and molecular function.

**Table 1:** The number of genes with significant differential expression considering *P*-value and LFC.

		**Significant genes**			
		***P* < 0.05**		***P* < 0.05, LFC >1, <−1**	
		**Down**	**Up**	**Down**	**Up**
**Accounting for genetic distance**	Sex Sea surface temperature Offshore versus far offshore	30	18	4	2
		1886	2393	-	-
		1585	1320	198	29
**Accounting for sea surface temperature**	Coastal versus offshore ecotypes	1620	1388	95	50
**Offshore females**	Pregnancy status	1	4	-	-

A ‘-’ indicates instances where there were no genes with an LFC greater or less than one. For sea surface temperature, a continuous variable, low LFCs are not unexpected as it represents a continuous rate of change and not an absolute change between conditions.

Weighted gene co-expression network analysis (WGCNA; Langfelder and Horvath, 2012, 2008) was used to construct co-expressed gene modules and identify networks of highly correlated genes. We used scale free topology to select a power setting and used blockwise modules to build gene modules. Blockwise modules were constructed with a minimum module size of 30 genes and a merge cut height of 0.35 for the full dataset. For the pregnancy related modules, we used the same minimum module size and a merge cut height of 0.55. Outliers were removed prior to analysis, as recommended by WGCNA, leaving 67 individuals for the larger module analysis and 14 individuals for examining pregnancy-related modules. We then tested for statistically significant relationships between co-expressed gene modules and experimental factors including ecotype, mean sea surface temperature, sex and pregnancy, followed by GO enrichment analysis of modules using TopGO. Though WGCNA analysis was conducted for all analyses, we will only be discussing gene modules related to sex and pregnancy. Additional details on all gene modules related to sea surface temperature, geographic location and ecotype are reported in supplemental documents (Results, Fig. S1).

**Figure 4:**
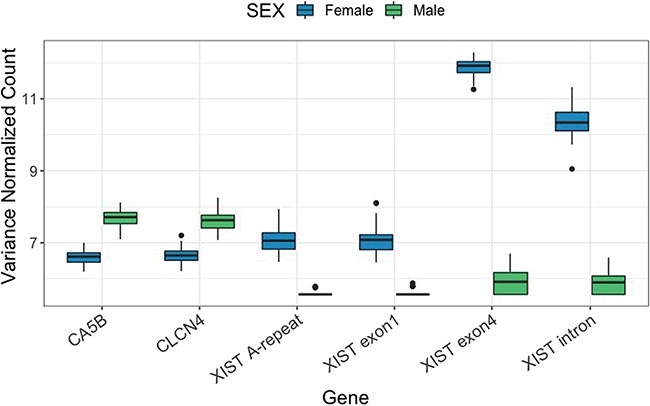
The six significantly differentially expressed genes between females and males with a fold-change greater than or less than two.

**Figure 5:**
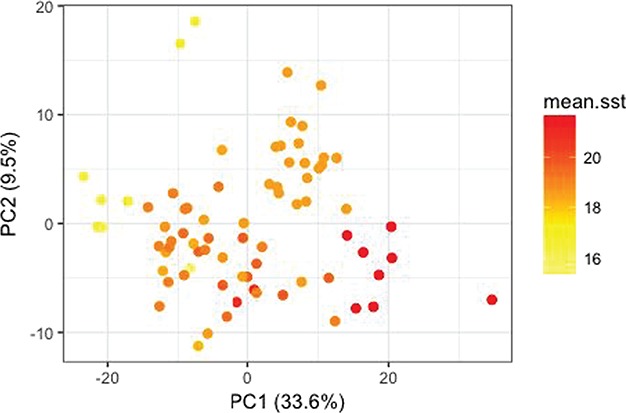
PCA of all samples for the set of genes that was differentially expressed with mean sea surface temperature (*N* genes = 4279).

## Results

We analysed differential expression of 20 698 putative genes across 75 *T. truncatus* individuals from the SCB. BUSCO assessment (Metazoa) of the genome-guided assembly found 92.9% complete BUSCOs, 5.9% fragmented BUSCOs and only 1.2% missing BUSCOs, indicating that our assembled reference skin transcriptome is reasonably complete. Each sample had an average of 12 million reads counted after trimming, quality filtering, alignment and annotation. Filtering out genes with low counts (i.e. those that were not detected in 45 or more individuals) resulted in 18 046 genes for differential expression analysis.

Genetic analysis of 14 715 SNPs identified putative population structure between the ecotypes analysed in this study (Fig. 2). PC1 distinguished the coastal ecotype from all other individuals, where offshore and far offshore groups primarily formed one genetic cluster (explaining 15% of the variation in the PCA). Four individuals within the far offshore group were clustered separately along PC2 in a PCA (only accounting for 3% of the variation), whereas the remaining far offshore individuals clustered strongly with the offshore ecotype. Fst calculations identified similar estimates of genetic differentiation between the coastal ecotype and each of the offshore ecotype and the far offshore group (as designated by sampling location), with or without the four outliers (Fst = 0.15). Fst between the far offshore and offshore groups was very small (Fst = 0.01). Here, we will be referring to these groups according to geographic location and ecotype. For geographic location, groups will be defined by three different sampling locations: coastal, offshore and far offshore (Fig. 2). For genetic ecotype, we combine all far offshore individuals with genetic similarities to the offshore individuals with this ecotype grouping.

**Figure 6:**
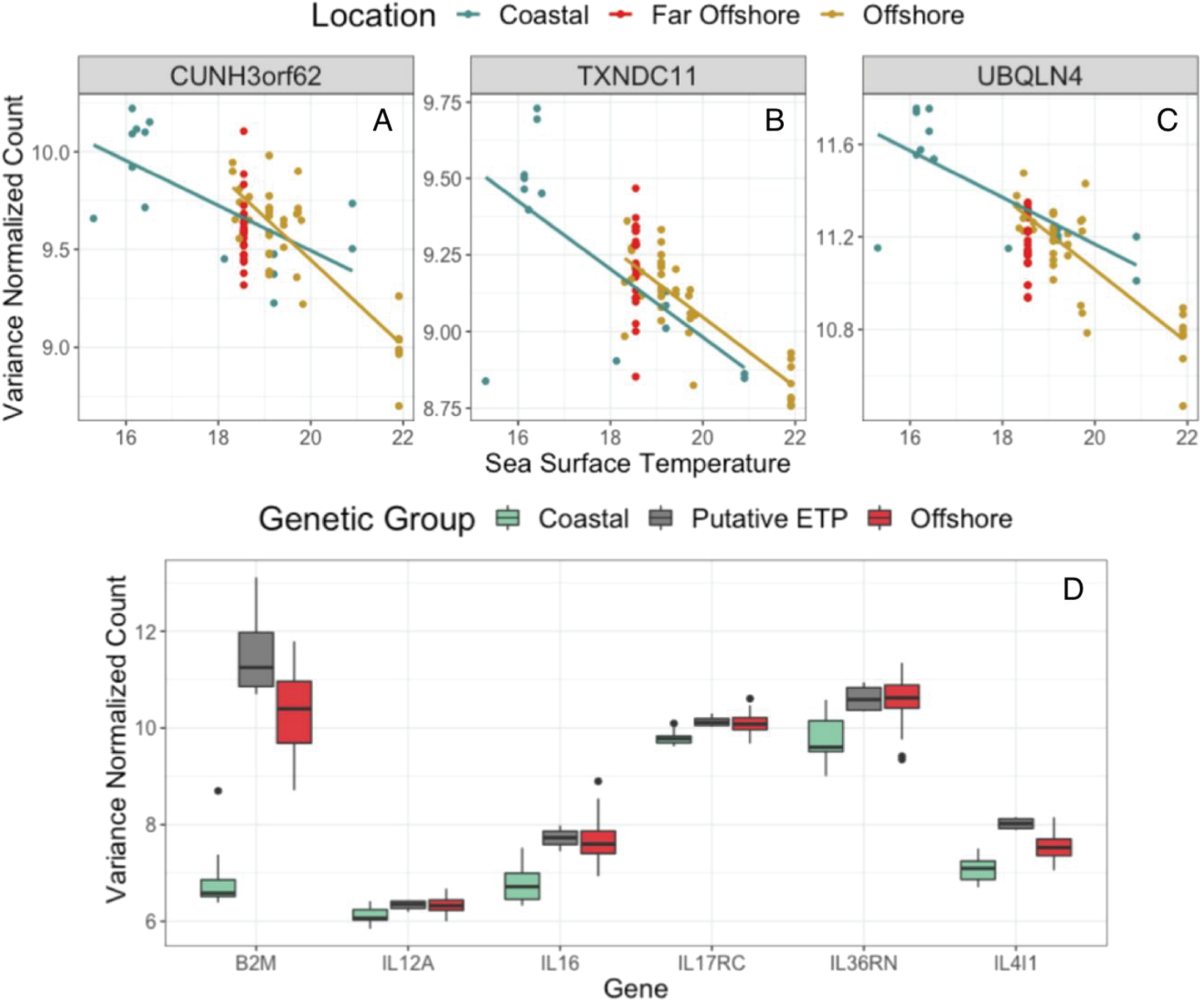
*Top*: The top three significant genes (i.e. the lowest *P*-values) associated with sea surface temperature from the model accounting for genetic distance. **A**) CUNH3orf62, a C3orf62 homologue. **B**) TXNDC11. **C**) UBQLN4. *Bottom*: Differentially expressed immune genes between ecotypes as designated by gene cluster: B2M, IL12A, IL16, interleukin 17 receptor C, interleukin 36 receptor antagonist and IL4I1.

### Differential expression related to sex and reproductive status

Forty-eight genes were significantly differentially expressed between sexes (Table 1). Most differences were subtle with only six genes having at least a 2-fold difference in expression levels between males and females (Fig. 4). Four of the six were non-coding RNAs related to the x inactive specific transcript (XIST) and were up-regulated in females. The other two significant genes, chloride channel protein 4 (CLCN4) and carbonic anhydrase 5B (CA5B), were up-regulated in males. Two modules of co-expressed genes were significantly correlated with sex. The grey60 module had the lowest *P*-value (Fig. S1, *r* = 0.98, *P* < 0.001) and was also correlated with XIST expression. This module was significantly enriched in X-linked genes according to a Fisher’s exact test where 2.6% of the genes in our transcriptome have homologues located on the human X chromosome compared to 20.53% of the 103 genes in the grey60 module (*P* < 0.001). Top GO terms enriched within with the grey60 module include interferon-alpha biosynthetic process, histone demethylase activity and ligand-gated ion channel activity (Fig. S1).

For pregnancy analysis, we analysed differential expression of 17 983 genes after filtering for low counts (i.e. those that were not detected in 11 or more individuals). We estimated 7 pregnancies and 11 non-pregnant individuals. We found one down- and four up-regulated genes (adjusted *P* < 0.05), none with a fold change >2 (Table 1). We identified four modules of co-expressed genes that were correlated with pregnancy, two of which were also correlated with blubber progesterone concentration (green and magenta modules, Table S2). The green module had the highest positive correlation with pregnancy (*r* = 0.62, *P* = 0.02). Notable GO terms enriched within the green module included cGMP catabolic processes, hypophysis morphogenesis and organic cyclic compound binding. The magenta module correlated most strongly with blubber progesterone (*r* = −0.59, *P* = 0.03). GO terms enriched within the magenta module include catabolic processes, blood vessel remodelling, U6 snRNP cellular components and hydrolase activity.

**Table 2:** The top three significant GO terms associated with differentially expressed genes relative to sea surface temperature, geographic location and genetic ecotype.

**Biological processes**		**Molecular function**	
**GO term**	***P-*value**	**GO term**	***P*-value**
**Sea surface temperature**			
Histone H4 acetylation	5.80E-05	Protein complex binding	9.70E-08
Cellular response to vascular endothelia...	0.00016	Macromolecular complex binding	2.20E-07
Extracellular structure organization	0.00017	Collagen binding	3.50E-07
**Geographic location: offshore versus far offshore**			
Regulation of response to stimulus	1.60E-05	Integrin binding	0.00012
Cellular response to glucose starvation	2.40E-05	Protein complex binding	0.00041
Collagen catabolic process	0.00012	Macromolecular complex binding	0.00062
**Genetic ecotype: coastal versus offshore**			
Defense response to other organism	8.00E-08	Ion binding	2.30E-05
Response to external biotic stimulus	6.60E-07	Lipopolysaccharide binding	0.002
Response to other organism	6.60E-07	Transforming growth factor beta binding	0.002

### Differential expression related to environmental variation

We found 4279 genes that were significantly differentially expressed with respect to mean sea surface temperature after accounting for genetic distance (Table 1). A PCA of these significant genes identified some stratification according to mean sea surface temperature (Fig. 5). The most highly significant genes related to sea surface temperature, CUNHorf62, thioredoxin domain-containing 11 (TXNDC11) and ubiquilin 4 (UBQLN4), were negatively related to sea surface temperature (Fig. 6). Top GO terms associated with sea surface temperature included histone H4 acetylation, vascular endothelial growth and extracellular structure (Table 2).

Due to the atypical sampling location of the far offshore group genetically related to the offshore ecotype, we investigated differential expression according to geographic location that were unrelated to genetic distance. By including differential expression by geographic location, we were able to investigate evidence of differential gene expression due to other environmental variables between genetically indistinguishable groups (i.e. within the offshore ecotype). We identified 2905 additional genes that were differentially expressed between the groups that were sampled in different geographic locations with minimal genetic distance: offshore and far offshore (Table 1). Of these differentially expressed genes, 227 were expressed >2-fold and 1713 (59% of all significantly different genes) were not correlated with sea surface temperature. GO terms associated with these differentially expressed genes included regulation of response to stimulus and cellular response to glucose starvation (Table 2).

### Differential expression between genetically distinct ecotypes

We detected 3008 genes differentially expressed between the genetically distinct coastal and offshore ecotypes, where the far offshore individuals that were genetically indistinguishable from the offshore ecotype were included within this grouping (Table 1). Of these, 145 genes had more than a 2-fold difference in expression between coastal and offshore groups. Beta-2-microglobulin (B2M) was the most significant differentially expressed gene (i.e. lowest *P*-value) between the coastal ecotype and the offshore ecotype, with lower expression in the coastal population (Fig. 6). Other significantly different genes included several interleukin genes related to immune response, cytokine signalling and cell redox: interleukin 12A (IL12A), interleukin 36 receptor agonist, interleukin 17 receptor C, interleukin 16 (IL16) and interleukin 4 induced 1 (IL4I1). Significantly enriched GO terms between coastal and offshore ecotypes were related to defense and immune response (Table 2).

## Discussion

We characterized baseline variation in transcriptome expression across two *T. truncatus* ecotypes within the SCB and related these to endogenous and exogenous variables. These results provide valuable information to inform future hypotheses regarding what factors affect the health and physiology of marine wildlife. Differential gene expression between males and females identified non-coding RNA transcripts related to gene inactivation on the X chromosome. While sex and pregnancy accounted for small differences in gene expression in *T. truncatus* skin, a larger portion of transcriptomic variation could be attributed to sea surface temperature. Differential expression explained by sea surface temperature included genes related to structural proteins.

Despite confounding factors due to sea surface temperature and genetic distance, major differences were apparent between geographic location and genetically distinct ecotypes. We observed transcriptomic variation between geographic groups that was not explained by genetic distance or sea surface temperature, indicating this group was potentially responding to exogenous environmental variation unaccounted for in this study (e.g. prey availability, pollution). Differential expression also highlighted potential differences in immune response between the coastal and offshore ecotypes. This research demonstrates the potential for transcriptomic analyses to improve our ability to understand how free-ranging marine mammals respond to their environment and to identify ecotypes that are more susceptible to environmental and anthropogenic disturbances.

### Differential expression related to sex and pregnancy status

Sex and reproductive status were a detectable but small source of transcriptomic variation among individuals. Though gene-by-gene statistical analyses revealed only a few genes were differentially expressed between sexes, analyses based on co-expressed modules indicated a network of genes that reside on the X-chromosome for which expression is diagnostic of sex. In particular, the four XIST-related non-coding RNAs are involved in silencing of the X chromosome in females and thus could have a large impact on expression of downstream genes associated with the X chromosome in females. Though our annotation has listed these as four separate genes, these may represent four isoforms of the same gene as there is only one XIST gene recognized in the human genome. The two genes more highly expressed in males (CLCN4 and CA5B) are both found on the X chromosome in humans. CLCN4 is also expressed more highly in male Gulf of Mexico (GOM) *T. truncatus*, suggesting that this pattern may be consistent across the species (Neely *et al.*, 2017). Gene module analysis identified a high proportion of X-linked genes compared to the rest of the transcriptome that were also highly correlated with XIST expression. This list includes CLCN4 and CA5B as well as the X-linked zinc finger protein ZFX, probable ubiquitin carboxyl-terminal hydrolase FAF-X (USP9X) and X-linked DEAD-box helicase 3 (DDX3X). High representation of x-linked genes in this module highlights potential X-linked gene network activity in wild cetacean skin, and the genes belonging to this module could facilitate sex identification in future cetacean studies.

Pregnancy is of particular interest in marine mammals as it is difficult to diagnose in free-ranging individuals (Kellar *et al.*, 2006) and can be an indicator of population health. Pregnancy also typically incurs elevated energetic costs (Reddy *et al.*, 1991) and could increase sensitivity of pregnant individuals to other stressors. Consistent with previous research (Van Dolah *et al.*, 2015), few expression differences were detected between presumed pregnant and non-pregnant individuals within offshore females, none of which were >2-fold. However, gene module analysis identified correlations between some gene modules and pregnancy status as well as blubber progesterone. GO terms associated with pregnancy modules involve organic compound binding, sterol binding and steroid binding, all of which may be indicative of changes in levels of steroid hormones present during pregnancy. Other enriched GO terms are related to catabolism, nucleosome binding, U6 snRNP activity and hypophysis (i.e. pituitary) morphogenesis. In order to incorporate inference of pregnancy into RNAseq analysis, it is likely important to include a larger sample size that will result in more robust gene modules; the sample size for this study is close to the minimum sample size recommended for WGCNA analysis. Our data suggest that sex is a more important endogenous factor influencing gene expression than pregnancy, a relevant finding for future RNAseq analyses on free-ranging marine mammals.

### Differential expression related to environmental variation

Though a small amount of transcriptomic variation was explained by endogenous factors, we identified a larger proportion of variation that could be attributed to exogenous factors. Indeed, skin condition is correlated with health and survival in North Atlantic Right Whales (Schick *et al.*, 2013), and skin biomarkers in cetaceans are a crucial tool for assessing ecological risk, for example from stress associated with contaminants, diet, pathogens and climate change (Godard-Codding and Fossi, 2018, Trego *et al*., 2019). Some of the expression differences that were observed appeared to stem from exposure to a range of sea surface temperatures. Similar to previous research (Neely *et al.*, 2017), we found genes related to skin function to be significantly related to sea surface temperature, indicating that the energy invested into skin structure may vary by thermal environment. Temperature is known to impact turnover of epidermal molt in marine mammals with cell turnover increasing as animals shift from cold to warm water (Aubin *et al.*, 1990). Oceanographic temperatures can also impact mammal distribution (Benson *et al.*, 2002), prey availability (Elorriaga-Verplancken *et al.*, 2016), the frequency of harmful algal blooms (HABs) (McCabe *et al.*, 2016) and the number and type of environmental pathogens (Burge *et al.*, 2014; Harvell, 2002; Shiah and Ducklow, 1994). UBQLN4, which was negatively related to sea surface temperature, is a gene related to regulation of protein catabolism and elimination of improperly folded proteins (Suzuki and Kawahara, 2016). TXNDC11 is also associated with the GO term for protein folding as well as cell redox. There was also significant enrichment in GO terms related to protein binding and ubiquitin-dependent protein catabolism. *T. truncatus* ecotypes in the GOM are known to exhibit high variation in skin gene expression profiles associated with seasonal variation where seasonal fluctuations are larger than observed in the SCB (Neely *et al.*, 2017; Van Dolah *et al.*, 2015). The temperature range of samples investigated in this study represents a much narrower thermal range (16–22°C), though this variable appears to contribute to variation within the skin transcriptome. These findings are consistent with changes in skin maintenance and structure associated with elevated temperatures in the sea surface environment, which could also be indicative of changes in organism level health.

Additional expression variation was detected between groups that were genetically similar but sampled from different geographic locations. Individuals sampled far offshore demonstrated different transcriptomic profiles compared to other offshore individuals sampled near the Channel Islands (Fig. 1). The majority of differentially expressed genes between these groups could not be explained by differences in sea surface temperature between these geographic locations. Rather, it is likely that these differences in gene expression reflect unmeasured variability within their environment not accounted for in this study. The far offshore sampling group included here was sampled in 2014 when anomalously warm conditions were observed in the SCB (Peterson *et al.*, 2017; Zaba and Rudnick, 2016). GO analysis suggests that these expression differences could be related to response to external stimuli and cellular response to glucose starvation, indicating potential nutritional stress. This could be linked to changes in primary production (Gómez-Ocampo *et al.*, 2017; Peterson *et al.*, 2017) and food availability (Elorriaga-Verplancken *et al.*, 2016) associated with abnormally warm oceanographic conditions. Given that this group was sampled in an anomalous year characterized not only by shifts in temperature but also several other factors, including prey distribution, it is possible that other environmental variables that were also indirectly influenced by shifts in oceanographic conditions were driving transcriptomic differences within this geographic group. Though sampling of wild cetaceans is inherently difficult, a more even distribution of sampling across years, ecotypes and temperatures throughout this region, in addition to the inclusion of additional environmental factors, could help disentangle different sources of transcriptomic variation and differentiate physiological differences due to temperature from those explained by other factors that vary within an ecotype.

### Differential expression between genetically distinct ecotypes

Even after accounting for sex and sea surface temperature, we detected unexplained transcriptomic variation between genetically distinct ecotypes. Differential gene expression between coastal and offshore *T. truncatus* within the SCB suggests differences in physiological status between groups, indicative of differential exposure to external variables not included in this study. One of the prominent differences we detected between ecotypes was a significant enrichment of genes associated with response to external stimuli such as pathogens or other external biotic stimuli. For example, we observed lower B2M expression, which is a component of the major histocompatibility complex (MHC) that is important for immune response. A similar pattern was observed in other immune related interleukin genes related to immune response, cytokine-signalling, inflammation and redox reactions. Our data indicate lower expression of immune genes in the coastal ecotype compared with the offshore ecotype.

There are several possible explanations for immune differences between these two genetically distinct groups. It is difficult to separate evolved differences between ecotypes from differential expression due to distinct environmental exposures. Evolved divergence of immune gene expression between these distinct ecotypes is possible, but there are also several other potential explanations for this pattern. Exposure to HABs may impose selective pressure on immune function within specific populations of bottlenose dolphins in the GOM with more frequent HAB exposure (Cammen *et al.*, 2015a, 2015b). Though the SCB does experience frequent HABs (Schnetzer *et al.*, 2007), we currently do not have data on whether algal bloom frequency and magnitude differ between these two areas and whether such differences would be substantial enough to contribute to selection on immune system genes in these ecotypes.

Beyond genetic divergence between stocks, differential expression of immune genes could also reflect physiologically induced differences following habitat-specific exposure to different environmental variables; these could include higher pathogen loads in the offshore ecotypes or a non-adaptive immune suppression in coastal individuals. Previously, the offshore ecotype was found to have greater incidence of skin lesions than the coastal ecotype (Bearzi *et al.*, 2009), which could be a sign of increased skin infection and immune response. Since temperature can influence skin structure and immunity in marine mammals, it could contribute to immune response in the skin. Neely *et al*. (2017) documented seasonal changes in the expression of genes associated with the MHC I Kegg pathways in *T. truncatus* in the GOM, though none were observed in this study. Furthermore, IL-16 is a pro-inflammatory cytokine typically associated with chemotaxis and viral response (Center *et al.*, 1997) and is not typically associated with temperature to our knowledge. Another explanation for this pattern includes immune suppression in the coastal ecotype, which could be related to increased exposure to anthropogenic contaminants that are known to inhibit immune responses. Regions of the SCB are polluted with high levels of polychlorinated biphenyls (PCBs; Blasius and Goodmanlowe, 2008; Chapman, 1996; Young *et al.*, 1991), which are known to suppress immune function in marine mammals (De Swart *et al.*, 1996; Ross *et al.*, 1996). Male individuals from the coastal ecotype that were sampled in the present study do have higher PCB loads than than those from the offshore ecotype (Trego *et al.*, 2019). Additional research is needed to confirm the source of physiological and genetic differences among *T. truncatus* in the SCB, particularly the source of significant differences in immune-related genes and the potential for interactive effects of exposure to temperature with other stressors.

### Conclusions

This study represents the one of first RNAseq analyses of marine mammals and temperature in the SCB and one of the few RNAseq analyses that have been conducted for wild cetaceans in the USA. Our research demonstrates the potential for transcriptomic analyses to inform our understanding of how wild cetaceans respond to their environment and to identify groups or populations that may be more vulnerable to environmental and anthropogenic disturbances. Our data support the hypothesis that temperature influences skin function in marine mammals through alteration of genes associated with structural proteins. Beyond localized skin maintenance, cetacean skin transcriptomes should reflect physiological responses to stressors at an organismal level, including the presence of systemic disease, contaminant exposure and poor nutrition. This information could provide invaluable insight into how stressors are impeding efforts to conserve marine mammal populations.

By incorporating genetic distance with RNAseq data, these findings provided further insight into genetic differentiation among offshore bottlenose dolphins in the Pacific and gene expression differences among geographic locations that were unexplained by genetic distance or sea surface temperature. Additional transcriptomic variation between two genetically distinct ecotypes was not accounted for by endogenous and exogenous factors measured here; notable differences in immune gene expression suggest that these ecotypes may differ in either their exposure to pathogens or to other immune-altering agents such as toxicants. These results highlight the utility of transcriptomic approaches to monitor health and physiology in free-ranging marine mammals and to identify key mechanisms that underlie how wild marine mammals respond to both natural and anthropogenic stressors.

## Supplementary Material

CP_Trego2018_SupplementaryData_Revision_coz018
