# Supplementary material for: Tracking transcriptomic responses to endogenous and exogenous variation in cetaceans in the Southern California Bight
Source: Conserv Physiol. 2019 May 15;7(1):coz018. doi: 10.1093/conphys/coz018 (PMC6518923; doi:10.1093/conphys/coz018)
Supplement: CP_Trego2018_SupplementaryData_Revision_coz018 [file cp_trego2018_supplementarydata_revision_coz018.docx]

**Supporting Information:**

**Results**

For the model that accounted for genetic distance, we identified 11 modules of co-expressed genes that were significantly correlated to sea surface temperature. Mean sea surface temperature was most strongly correlated with the yellow module (r = 0.79, p < 0.001) which was enriched for GO terms including carbohydrate phosphorylation, peroxisome fission, and regulation of cytoplasmic translation (Figure S1). Nine gene modules were significantly correlated with geographic location. The red module was most highly correlated with location (r = -0.79, p < 0.001) and was enriched for GO terms including anion transport, multicellular organismal reproductive processes, and substrate specific transporter activity (Figure S1). In the model comparing genetically distinct ecotypes, we found 11 modules of co-expressed genes that were significantly correlated to genetic ecotype, where the red module was most strongly correlated (r = -0.86, p < 0.001). The red module was highly linked with B2M expression.

**Figure S1:** A heatmap and the gene ontology terms associated with each WGCNA gene modules. The heatmap represents a correlation matrix for each gene module compared to four variables assessed in this study: location, sex, sea surface temperature (SST), and ecotype. The color represents the correlation coefficient where red is positive and blue is negatively correlated. Each box provides the corresponding correlation coefficient and p-value in parentheses for each comparison.

**
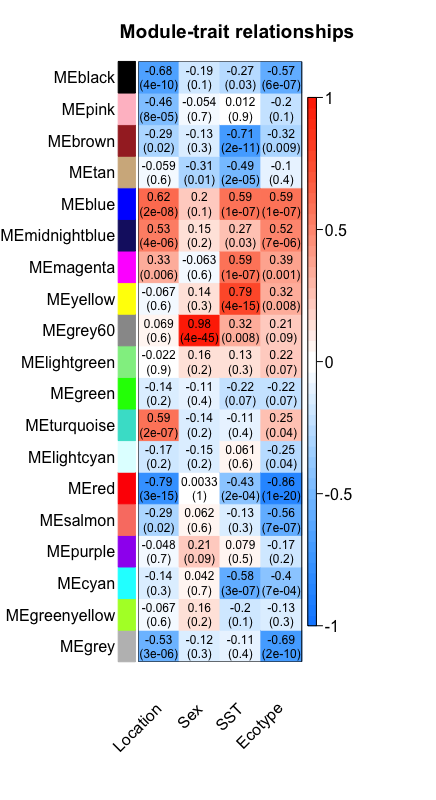
**

**Table S1** The blubber progesterone concentration data for each individual from the offshore ecotype that were considered for pregnancy transcriptome analysis. The pregnancy status was determined according to cutoffs derived from derived from data in Kellar *et al*. (2017).

| **Ecotype** | **Year** | **Blubber Progesterone (ng/g)** | **Pregnancy Status** |
| --- | --- | --- | --- |
| **O** | **2012** | **0.34** | **NP** |
| **O** | **2015** | **0.42** | **NP** |
| **O** | **2015** | **0.44** | **NP** |
| **O** | **2015** | **0.53** | **NP** |
| **O** | **2012** | **0.60** | **NP** |
| **O** | **2015** | **0.64** | **NP** |
| **O** | **2014** | **0.65** | **NP** |
| **O** | **2015** | **0.83** | **NP** |
| **O** | **2015** | **1.80** | **NP** |
| **O** | **2015** | **8.55** | **NP** |
| **O** | **2015** | **9.23** | **NP** |
| **O** | **2014** | **26.11** | **U** |
| **O** | **2015** | **26.22** | **U** |
| **O** | **2013** | **29.59** | **U** |
| **O** | **2014** | **32.37** | **U** |
| **O** | **2014** | **48.35** | **P** |
| **O** | **2015** | **62.62** | **P** |
| **O** | **2015** | **68.43** | **P** |
| **O** | **2015** | **73.51** | **P** |
| **O** | **2014** | **83.03** | **P** |
| **O** | **2014** | **102.57** | **P** |
| **O** | **2013** | **420.69** | **P** |

**Table S2** Gene ontology terms associated with WGCNA gene modules that were highly correlated with pregnancy (green module) and both pregnancy and progesterone levels (magenta modules).
